# Jenny Pronczuk de Garbino: A Global Champion for Children’s Health

**DOI:** 10.1289/ehp.1408293

**Published:** 2015-03-01

**Authors:** Philip J. Landrigan, William A. Suk

**Affiliations:** 1 Department of Preventive Medicine, and; 2 Arnhold Global Health Institute, Icahn School of Medicine at Mount Sinai, New York, New York, USA; 3 National Institute of Environmental Health Sciences, National Institutes of Health, Department of Health and Human Services, Research Triangle Park, North Carolina, USA

On 20 September 2010, the children of the world, and all of us who work in children’s environmental health and pediatric toxicology, lost a beloved friend and global champion with the untimely death of Jenny Pronczuk de Garbino at 63 years of age. Pronczuk was a physician and, for many years, a medical officer in the Department of Public Health and Environment of the World Health Organization (WHO). She was the founder and, for more than a decade, the charismatic and inspirational leader of the WHO Initiative in Children’s Environmental Health. Her work improved the lives of children in all regions of the world.

Pronczuk was born in Uruguay of Ukrainian parents. She grew up in Montevideo and the wild countryside of rural Uruguay, which she loved deeply. She was trained as a physician at the School of Medicine of the Universidad de la República in Uruguay, completing her postgraduate studies in clinical toxicology and occupational health. She undertook further clinical training at the Université de Paris, Lariboisière–St Louis, and then at Baylor College of Medicine in Houston, Texas, through a Fulbright Scholarship. On completion of her training, she was appointed Head Professor of Clinical Toxicology and Director of the National Poisons Centre in Montevideo.

Pronczuk joined WHO in 1991, working initially in WHO’s International Programme on Chemical Safety. In this position, she facilitated the establishment of poison control centers worldwide, developed training programs in medical toxicology throughout the developing world, and published numerous authoritative articles.

In 1999, Pronczuk established the WHO Task Force for the Protection of Children’s Environmental Health, which became the WHO Initiative in Children’s Environmental Health. In 2002, WHO Director-General Gro Harlem Brundtland formally launched the initiative during the World Summit on Sustainable Development. The goal of the initiative was to prevent children’s disease and disability associated with environmental threats to health. It was here that Pronczuk truly found her life’s work.

Over the next decade, Pronczuk worked tirelessly to build the WHO Initiative in Children’s Environmental Health. She was an extraordinary leader with a truly amazing ability to bring people together to work joyfully to protect the health of children. She convened a series of landmark conferences and consultations in Bangkok, Thailand (2002); Mar del Plata, Argentina (2005); Buenos Aires, Argentina (2005); and Busan, Republic of Korea (2009). These conferences established priorities for action, built commitments to national and international programs, and energized pediatricians and medical societies. They led to the creation of a global network of Centers for Excellence in Children’s Environmental Health and also built collaborations among prospective birth cohort studies around the world examining environmental influences on children’s health and development. Important proclamations at these conferences provided critical guidance to the global development of children’s environmental health: the Bangkok Statement on Children’s Environmental Health (Suk et al. 2003; World Health Organization 2002); the Declaration of Mar del Plata, in which Ministries of Health and national pediatric societies in seven countries of the Southern Cone of South America pledged to make the protection of children against environmental threats to health a high national priority (Health and Environment Ministers of the Americas 2005); and the Busan Pledge for Action on Children’s Health and the Environment (Third WHO International Conference on Children’s Health and the Environment 2009), which called for a global plan of action to improve children’s environmental health in countries around the world (Gavidia et al. 2010).

Pronczuk led the development of an extraordinary body of work that created a firm intellectual foundation for children’s environmental health in countries around the world. She led the development of a training tool—the WHO Training Package for Health Care Providers, specifically for children’s environmental health (WHO 2011)—and implemented training workshops based on this tool. She oversaw construction of the “Green Page” for environmental health history taking (WHO 2012). She spearheaded the incorporation of a children’s environmental health module, based on the Green Page, into WHO’s Integrated Management of Childhood Illnesses (WHO 2015). She led the development of environmental health indicators for use in countries around the world (WHO 2009). Pronczuk promoted cooperative research, for example, in childhood asthma, arsenic exposure in pregnant women and their children in Thailand, and asthma and air pollution in urban areas in India. She also stimulated important contributions to the Global Burden of Disease project, showing, for example, that lead is responsible for approximately 1% of the global burden of disease (Fewtrell et al. 2004) and that toxic chemicals are responsible for 5.7% (Prüss-Ustün et al. 2011).

Pronczuk built strong proactive partnerships with nongovernmental organizations such as the International Society of Doctors for the Environment, the International Conference on Children’s Health and the Environment, and the Nurses Network. She worked with the United Nations Environment Programme to remove lead from gasoline worldwide. Pronczuk also worked closely with colleagues at the National Institute of Environmental Health Sciences (NIEHS), especially with Bill Suk. She was for many years the co-principal investigator of a cooperative agreement between NIEHS and WHO. She collaborated with scientists at NIEHS on initiatives to improve children’s health and to build effective partnerships worldwide. Pronczuk was remembered at NIEHS for her contagious laugh and her passion for making a difference in children’s lives all over the world.

In 2008, Pronczuk was recognized by the U.S. Environmental Protection Agency as the International Children’s Environmental Health Champion because of her outstanding efforts and commitment to advancing children’s environmental health issues around the world.

The memory and life’s work of Pronczuk were honored on 22–24 March 2012 at an International Scientific Conference on Environmental Health in the Political Agenda, held in Montevideo, Uruguay, her birthplace. The conference was convened by the Faculty of Medicine of the University of the Republic of Uruguay, the Pan American Health Organization, WHO, the Collegium Ramazzini, and NIEHS. The goal was to address critical and emerging issues in children’s environmental health, especially focusing on Latin America and the Caribbean. The conference looked to the future while celebrating Pronczuk’s extraordinary achievements. The first Jenny Pronczuk Award for Research and Intervention in Children’s Environmental Health and Toxicology was awarded at the conference to Maria Gabriela Rovedatti and colleagues for their paper, “Impact of Pesticide Environmental Exposure in the Triad Mother–Placenta–Fetus and Preventive Actions,” subsequently published (Cecchi et al. 2012). A report from the conference titled “Children’s Health in Latin America: The Influence of Environmental Exposures” (LaBorde et al. 2015) is published in this issue of *Environmental Health Perspectives*.

Pronczuk’s great legacy lies in the programs she built, the friends and colleagues she brought together, the young physicians and scientists whom she inspired, and the lives of the millions of children in countries around the world whose health she steadfastly protected.
